# Integrated Transcriptome Analysis of Long Noncoding RNA and mRNA in Developing and Aging Mouse Retina

**DOI:** 10.1038/s41597-023-02562-9

**Published:** 2023-09-23

**Authors:** Kangjie Kong, Peiyuan Wang, Zihong Xie, Lu Wang, Jiaxuan Jiang, Yaoming Liu, Shaolin Du, Jingwen Jiang, Yunhe Song, Fengbin Lin, Wei Wang, Xiuli Fang, Zhuoxing Shi, Xiulan Zhang, Shida Chen

**Affiliations:** 1 State Key Laboratory of Ophthalmology, Zhongshan Ophthalmic Center, Sun Yat-sen University, Guangdong Provincial Key Laboratory of Ophthalmology and Visual Science, Guangdong Provincial Clinical Research Center for Ocular Diseases, Guangzhou, 510060 China; 2 Joint School of Life Sciences, Guangzhou Medical University and Guangzhou Institutes of Biomedicine and Health (Chinese Academy of Sciences), Guangzhou, 510060 China; 3 The Second Affiliated Hospital of Guangzhou University of Chinese Medicine, Guangzhou, 510060 China; 4 Dongguan Tungwah Hospital, Dongguan, 523000 China

**Keywords:** Ageing, RNA sequencing

## Abstract

Mice have emerged as a widely employed model for investigating various retinal diseases. However, the availability of comprehensive datasets capturing the entire developmental and aging stages of the mouse retina, particularly during the elderly period, encompassing integrated lncRNA and mRNA expression profiles, is limited. In this study, we assembled a total of 18 retina samples from mice across 6 distinct stages of development and aging (5 days, 3 weeks, 6 weeks, 10 weeks, 6 months, and 15 months) to conduct integrated lncRNA and mRNA sequencing analysis. This invaluable dataset offers a comprehensive transcriptomic resource of mRNA and lncRNA expression profiles during the natural progression of retinal development and aging. The discoveries stemming from this investigation will significantly contribute to the elucidation of the underlying molecular mechanisms associated with various retinal diseases, such as congenital retinal dysplasia and retinal degenerative diseases.

## Abstract (summary)

**Table:** 

**Design Type(s)**	organism development and aging design • transcriptional profiling by RNA sequencing design
**Measurement Type(s)**	long noncoding RNA and mRNA profiling assay
**Technology Type(s)**	illumina RNA sequencing
**Factor Type(s)**	life cycle stage
**Sample Characteristic(s)**	*Mus musculus* • retina

## Backgroud & Summary

Mice serve as an invaluable model for investigating retinal diseases due to their resemblance to the human retina in terms of structure and developmental patterns^1–3^. Upon reaching the neonatal stage, mice undergo the pivotal event of eye opening and light perception, during which retinal progenitor cells differentiate into distinct cell types, leading to the formation of retinal layers^4^. The maturation of synapses, dendrites, and cell junctions contributes to the progressive development of the retina, ultimately culminating in the specification and differentiation of retinal ganglion cells (RGC), Müller cells, bipolar cells, horizontal cells, amacrine cells, and rod/cone photoreceptor cells. The precise orchestration of this developmental process relies on the intricate regulation of transcription factors (TFs)^5–7^. Exploring the transcriptome networks involved in the differentiation of retinal progenitor cells offers valuable insights into the developmental patterns of the retina and the broader central nervous system. Furthermore, identifying retinal aging can be challenging through current clinical imaging technology in human and pathological examination of animal tissue, especially in the early old stage. Previous studies have highlighted various hallmarks of cellular aging, including nuclear abnormalities, telomere attrition, mitochondrial dysfunction, genetic instability, and epigenetic alterations^8^. Transcriptome analysis presents a powerful approach to comprehensively investigate the genetic and epigenetic changes associated with the aging process and age-related retinal disorders.

Long noncoding RNAs (lncRNAs) are a class of RNA characterized by their length longer than 200nt and lack of protein coding function^9^. In comparison to messenger RNA (mRNA), most lncRNAs are typically less annotated and their functions are largely unexplored. However, lncRNAs may bind to microRNAs or TF, which can regulate the expression of downstream genes^10^. In this way, lncRNAs may play crucial roles in multiple biological and pathological processes such as retinal cell differentiation, maintain retinal structure and homeostasis, retinal aging and degeneration in mouse models^11–14^. Chen *et al*.^15^ revealed 2600 lncRNAs in developmental mice retina from embryonic period to adult period using whole transcriptome sequencing. Wan *et al*.^16,17^ characterized 5404 lncRNA genes and 940 intergenic lncRNAs in retinas from the embryonic day of 12.5 to the neonatal day of P28. However, our understanding of the integrated expression profiles of lncRNAs and mRNAs throughout the various stages of retinal development, particularly during the aging process, remains limited.

In this study, we utilized a total of 18 mouse retina samples spanning six distinct developmental periods, encompassing the transition from neonatal to aging. These samples were subjected to integrated lncRNA and mRNA sequencing using Illumina paired-end 150 bp (PE150) sequencing. Critical steps in the experimental pipeline, such as the evaluation of raw and clean reads, correlation and clustering of sequencing libraries by gene and transcript quantification, assembly and validation of lncRNA/mRNA transcripts, and other bioinformatic analyses, received high scores and demonstrated exceptional reliability. This transcriptome dataset represents a highly valuable resource that will significantly contribute to diverse fields of retinal research, including explorations into retinal biomarker discovery, gene therapy investigations, and the advancement of retinal organoid models.

## Methods

### Ethical approval

This study received approval from the ethics committee of the institutional review board at Zhongshan Ophthalmic Center. All procedures were conducted according to the ethical standards of the research committee. Male C57BL/6 mice were purchased from Jiesijie Laboratory, Shanghai, China.

**Fig. 1:**
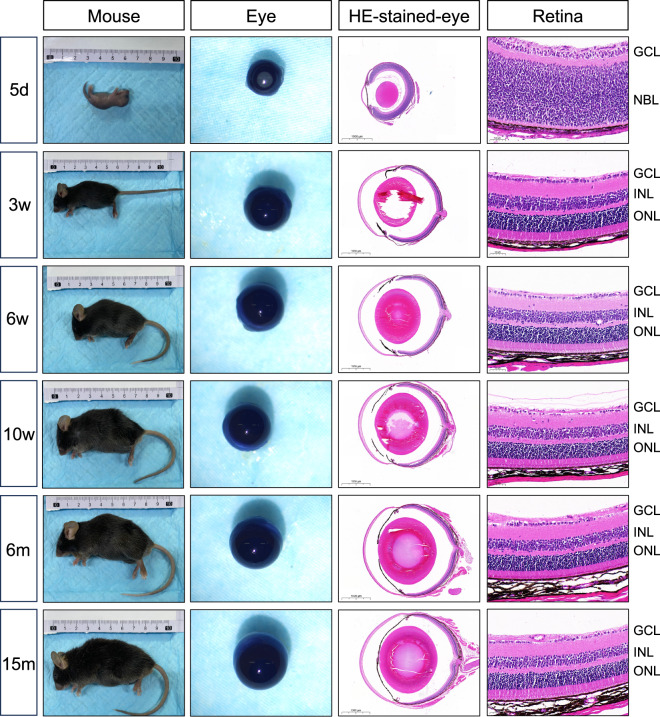
Overview of six distinct stages of developing and aging in mice eyes and retinas. The body length of the mice and eye size gradually increased from CE to OP. In HE stained retinal sections, after retinal progenitor cells differentiation to multiple retinal cells, retina with different layers were gradually formed from CE to SP. CE: close eye period; SP: suckling period; PP: puberty period; AP: adult period; MP: middle period; OP: old period; d: days; w- weeks; HE: hematoxylin and eosin; GCL: ganglion cell layer; NBL: neuroblast layer; INL: inner nuclear layer; ONL: outer nuclear layer.

The postnatal time points selected for the study, including 5 days, 3 weeks, 6 weeks, 10 weeks, 6 months, and 15 months, corresponded to distinct developmental stages, including the neonatal stage or close eye (CE) period, suckling period (SP), puberty period (PP), adult period (AP), middle period (MP), and old period (OP), respectively (Fig. 1).

### Neuro-retina collection and histology

All mice were anesthetized through the intraperitoneal injection of 0.3% pentobarbital sodium, and then sacrificed for analysis. The eyes were enucleated and washed with PBS. Cornea was dissected along with limbus before removing lens and iris. Then neuro-retina was separated from RPE-choroid-sclera and washed with PBS to remove residual pigment (Supplementary Fig. 1). Neuro-retina was placed in 1.5 ml EP tubes and stored at −80 °C.

The eyes were enucleated and fixed in 4% paraformaldehyde for 24 hours. After immersion in 70% alcohol, the eyes were embedded in paraffin and sectioned at 5-μm intervals in the sagittal plane. Sections through the optic papilla were collected and stained with hematoxylin and eosin (HE). HE stained sections were recorded and using CaseViewer software (v2.2). The area 250–300 µm from the optic nerve was intercepted and magnified as a typical picture of retina.

### Total RNA isolation and qualification

Total RNA was extracted from retina samples by using TRIzol reagent (Life Technologies, Carlsbad, CA, USA) according to the manufacturer’s instructions, including treatment with DNase. Bilateral retinas from one mouse were pooled as a sample in all groups. RNA degradation and contamination was monitored on 1% agarose gels with 180 V for 16 min. RNA purity was checked using the NanoPhotometer® spectrophotometer (IMPLEN, CA, USA), and samples with an optical density 260/280 and 260/230 ratio between 1.8 and 2.1 were used. RNA integrity was assessed using the RNA Nano 6000 Assay Kit of the Bioanalyzer 2100 system (Agilent Technologies, CA, USA) to calculate an RNA Integrity Number (RIN). Only RNA samples with a RIN > 8 were used for sequencing analysis. A total amount of 3 µg RNA per sample was used as input material for the RNA sequencing library preparations.

### Strand-specific library construction and qualification

**Fig. 2:**
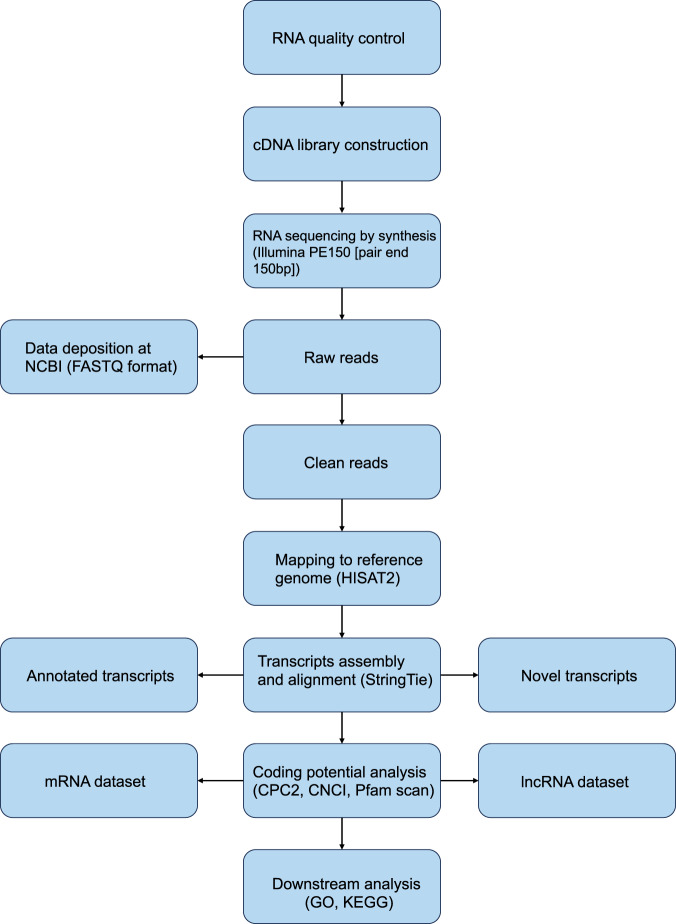
Workflow of RNA sequencing and analysis.

The workflow of RNA sequencing was shown (Fig. 2). After removing ribosomal RNA (rRNA), total RNA was break into short fragments of 250–300 bp. The fragmented RNA was used as a template and random oligonucleotides were used as primers to synthesize the first strand of cDNA, then RNA templates were degraded with RNase H. The second strand of cDNA was synthesized in DNA polymerase I system with the first strand of cDNA as templates and dNTPs (N = A, C, G, U) as raw materials. The double-stranded cDNA got through end repair, adding A-tail and adaptors, and the cDNAs of about 200 bp were selected using AMPure XP beads (Beckman Coulter, Beverly, USA). The second strand of the cDNAs containing dUTPs were digested using 3 μl USER enzyme (NEB, USA) at 37 °C for 15 min followed by 5 min at 95 °C, and finally PCR was performed to amplify and construct the library.

After library construction, initial quantification was performed using Qubit 2.0 and dilute the library to 1.5 ng/ul, followed by detection of the insert size of the library using Agilent 2100. Then the effective concentration of the library (>3 nM) was accurately quantified using qRT-PCR.

### Illumina sequencing

After passing the library qualification, the library was pooled and sequenced by Illumina PE150 (paired-end 150 bp), which referred to high-throughput paired-end sequencing of 150 bp at each end, and the insert cDNA was the unit directly sequenced in the constructed library. The basic principle of sequencing was sequencing by synthesis. Four fluorescently labeled dNTPs (N = A, C, G, T), DNA polymerase and splice primers were added to the sequencing flow cell for amplification. The sequence information of the fragments was obtained by capturing the fluorescence signal and converting into sequencing peaks.

### Reads filtering and mapping

The raw image files are converted into sequenced reads by CASAVA (v1.8) base identification and stored in FASTQ format. Raw data (raw reads) of FASTQ format were firstly processed through in-house perl scripts. During this process, clean data (clean reads) were obtained after removing low-quality reads (>50% of bases with sQ < 5) and reads containing adapter or ploy-N (>10% of uncertain base) from raw data. Reference genome and gene model annotation files were downloaded from genome website directly. Index of the reference genome was built and clean reads were aligned to the reference genome using HISAT2 (v2.0.5)^18^, which might generate a precise mapping result for junction reads.

### Transcripts assembly and classification

Transcripts were assembled using StringTie (v1.3.1)^19^. After assembly, transcripts with length >200 bp and exon number ≥2 were selected. Then, these transcripts were aligned to annotated database using Cuffcompare (v2.2.1)^20^ and classified into annotated transcripts and novel transcripts. The mainstream coding potential analysis methods (CPC2, v3.2.0; Pfam scan, v1.3; CNCI) were integrated to performed the screening of coding potential. The next selection and naming of candidate novel lncRNA was referred to the HGNC (The HUGO Gene Nomenclature Committee)^21^ guideline criteria. In order to further validate the accuracy of our transcript classification approach, we compared the transcript length, exon number, and open reading frame length of the novel lncRNAs with those of mRNAs. Following the aforementioned procedures, the transcripts were categorized into annotated mRNAs, annotated lncRNAs, novel mRNAs, and novel lncRNAs.

### LncRNA/mRNA transcript quantification

We utilized StringTie (v1.3.1) for quantifying transcript-level expression. StringTie employs advanced algorithms to effectively reconstruct transcript structures and estimate their abundance using RNA-seq reads aligned to a reference genome. We inputted spliced alignments in coordinate-sorted BAM file and obtained a GTF output containing assembled transcript structures, along with their corresponding expression levels reported as FPKM (Fragments Per Kilobase of transcript sequence per Millions base pairs sequenced). These FPKM values were utilized for downstream analyses.

### Differential expression analysis

Differential expression analysis between any two groups was performed using the EdgeR R package (v3.12.1)^22^. EdgeR was used to adjust read counts prior through one scaling normalized factor for each sequenced library. This package utilizes a statistical framework based on the negative binomial distribution to determine differential expression. The resulting P-values were adjusted using the Benjamini and Hochberg’s method to control the false discovery rate. Genes and transcripts with an adjusted P-value < 0.05, as determined by EdgeR, were considered as differentially expressed.

### GO and KEGG enrichment analysis

We utilized the clusterProfiler R package (v4.6.2)^23^ to conduct statistical enrichment analysis of differentially expressed genes. This analysis included Gene Ontology (GO) enrichment analysis^24^ and Kyoto Encyclopedia of Genes and Genomes (KEGG) pathway analysis^25^. GO enrichment analysis provided insights into the biological processes, cellular components, and molecular functions associated with the differentially expressed genes. KEGG pathway analysis was employed to identify relevant signaling pathways. GO terms with a corrected P-value below 0.05 were considered significantly enriched by the differentially expressed genes.

### Quantitative real-time PCR (qRT-PCR)

After isolation of total RNA, complementary DNA was synthesized with Evo M-MLV RT Kit (AG11707, Accurate Biotechnology, Hunan, China) and amplified with SYBR® Green Premix Pro Taq HS qPCR Kit (AG11701, Accurate Biotechnology) on CFX96 real-time PCR system (Bio-Rad), using GAPDH as an internal reference. Then, relative expression was calculated using the 2^-ΔΔCT^ formula. Primers of the selected lncRNAs and mRNAs were synthesized by Sangon Biotech, Shanghai, China, and the sequences were as follows:

lncRNA-otx2os1-F: 5′-GCAACTCTGTCCGCTTGTTG-3′;

lncRNA-otx2os1-R: 5′-CCCTAGACGTCTGCAAAGCA-3′;

GAPDH-F: 5′-TGTGTCCGTCGTGGATCTGA-3′;

GAPDH-R: 5′-TTGCTGTTGAAGTCGCAGGAG-3′.

### Fluorescence *in situ* hybridization (FISH)

FISH staining was completed according to the instruction of RNA FISH Detection Reagent kit (Servicebio, Wuhan, China) as previously described^26^, and finally photographed using a confocal microscopy (Leica, Wetzlar, Germany). Sequencing of lncRNA-otx2os1 probe mix were as follows:

5′-GTATCACGAGCAAAGACAAGCCCTG-3′;

5′-AAGAGCAATTTTGCAACTTTTCCAG-3′;

5′-CGGACAGAGTTGCTTATTCTCAGGG-3′;

5′-ACACATCCTGAGCCCCTAGACGTCT-3′;

5′-GAGTGTTCTTTTGCAGGGCACATAA-3′

## Data Records

Raw reads of all samples were deposited in the Sequence Read Archive (SRA) of the National Center for Biotechnology Information (NCBI) as FASTQ files with SRP accession number, SRP445437^27^. The files of reference mapping, assembly and lncRNAFilter, quantification, differential expression analysis, GO and KEGG enrichment analysis were also deposited in figshare^28^ (10.6084/m9.figshare.23614980.v1).

## Technical Validation

### RNA-Seq raw data quality and filtering

**Fig. 3:**
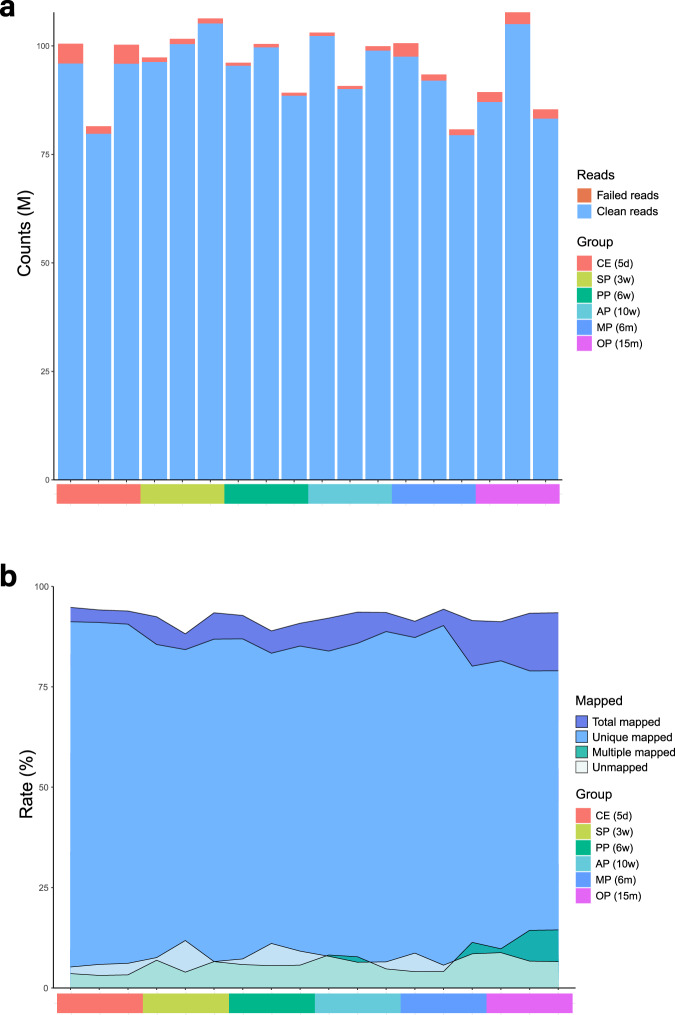
Raw reads filtering and clean reads mapping. (**a**) Counts of clean reads and failed reads in raw reads of 18 samples. (**b**) Mapping rate to mouse reference of clean reads of 18 samples.

Quality control and reads statistics were shown (Supplementary Table 1). Clean reads/ raw reads (mean ± SD) were 98.17% ± 1.17%, error rate was 0.013% ± 0.005% (Fig. 3a). Raw_bases(G) and Clean_bases(G) were 14.37 ± 1.17 and 14.11 ± 1.16. Q20, Q30 and GC content of the clean data were 97.21 ± 0.53%, 93.04 ± 1.15% and 47.66 ± 1.50%. The normally distributed GC content indicated the sequencing data were not contaminated.

### Clean reads mapping

The clean reads were aligned to the mouse genome (GRCm38) provided by the Genome Reference Consortium. The total mapped reads for all samples exceeded 90%, except for PP2 and SP2, which were 88.92% and 88.19% respectively. Uniquely mapped reads for all samples were over 80%, except for OP2 and OP3, which were 78.97% and 78.99% respectively. The percentage of reads mapped to exonic or intronic regions of the genome exceeded 90% for all samples, except for OP1 and MP3, which were 88.82% and 83.02% respectively (Fig. 3b, Supplementary Table 2).

### Reproducibility of biological replicates

**Fig. 4:**
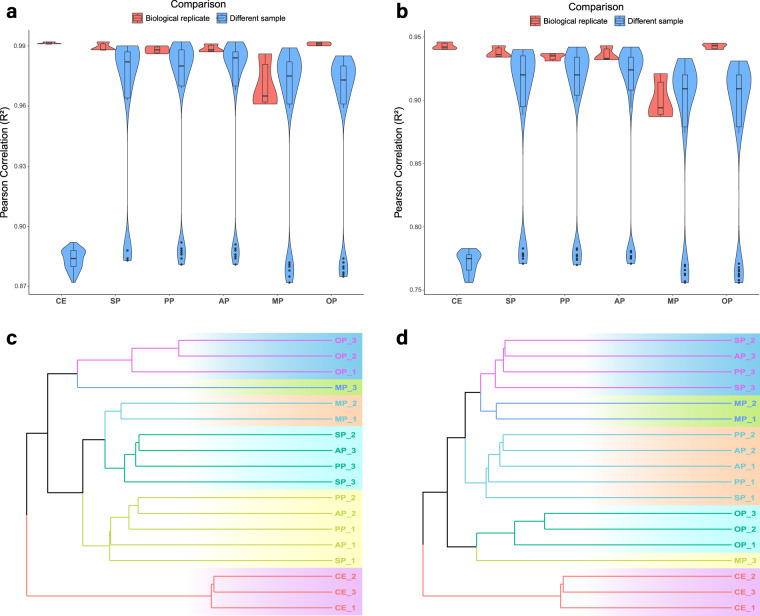
Pearson correlation analysis and cluster analysis between samples in gene and transcript level. Pearson correlation analysis between biological replicates both got high scores (**a**) in gene level and (**b**) in transcript level, CE group showed great difference from other samples. Cluster analysis showed that CE and OP groups were independent from other groups (**c**) in gene level and (**d**) in transcript level. CE: close eye period; SP: suckling period; PP: puberty period; AP: adult period; MP: middle period; OP: old period.

To evaluate the reproducibility of biological replicates, we computed Pearson’s correlation coefficients for FPKM expression at both the gene and transcript levels among all pairwise combinations of the 18 samples. At the gene level, all correlation scores between samples within the same developmental period group exceeded 0.96 (Fig. 4a). At the transcript level, all correlation scores between samples within the same developmental period group were greater than 0.93, except for MP3 vs MP1 and MP3 vs MP2, which were 0.894 and 0.887, respectively (Fig. 4b). Cluster analysis of FPKM expression at the gene and transcript levels revealed that the CE and OP groups formed distinct clusters independent from other groups at the gene level (Fig. 4c) and at the transcript level (Fig. 4d). The SP, PP, AP, and MP groups clustered together due to their relatively high similarity in sample profiles.

### LncRNA and mRNA transcriptome

**Fig. 5:**
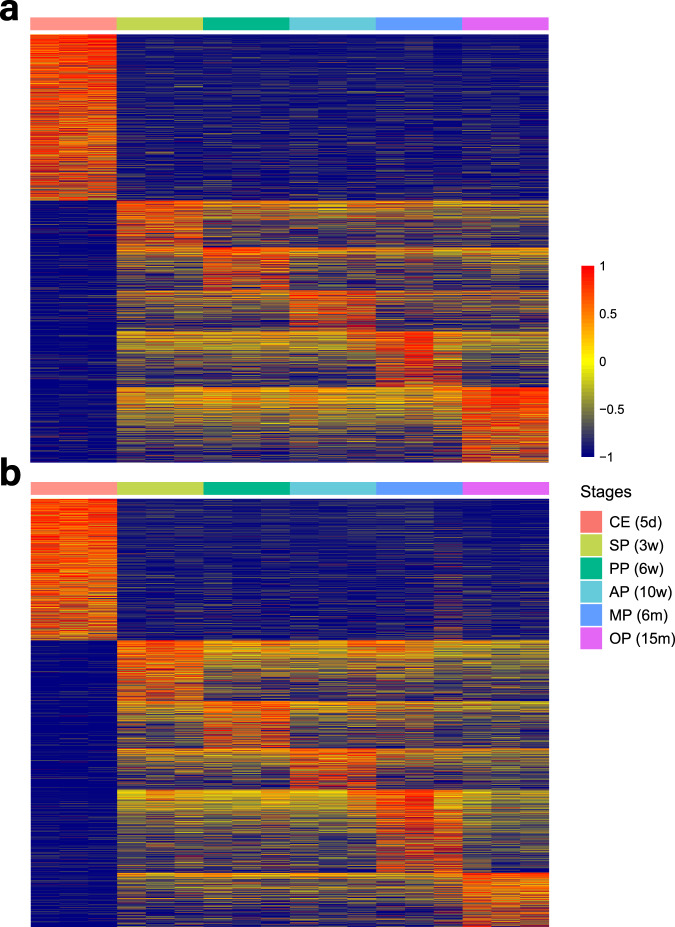
Heatmaps of differentially expressed genes in 18 samples. Heatmaps showed the overview of quantification of (**a**) differentially expressed mRNA genes and (**b**) differentially expressed lncRNA genes in 18 samples.

**Fig. 6:**
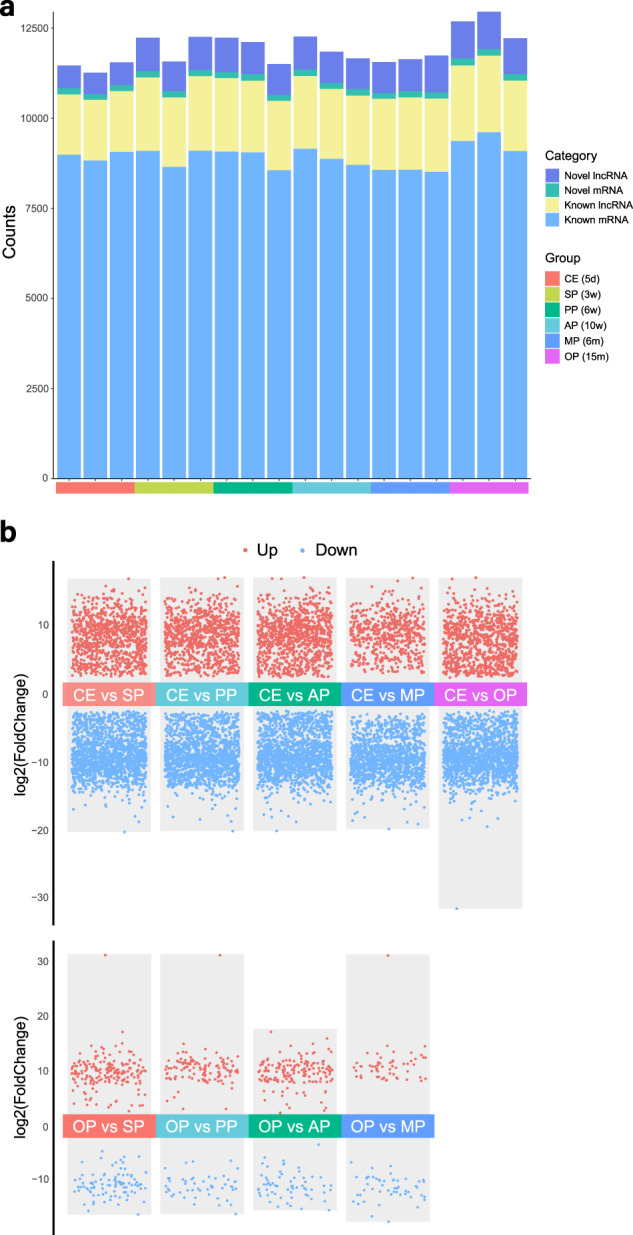
Differentially expressed transcripts between two groups. (**a**) Differentially expressed transcripts in each sample were classified as novel lncRNA, novel mRNA, known lncRNA and known mRNA. (**b**) Vocalno plots showed that numbers of differentially expressed lncRNA transcripts in CE vs other groups were larger than that in OP vs other groups.

In gene-level expression analysis, we detected a total of 11,222 lncRNA genes (Fig. 5a) and 18,809 mRNA genes (Fig. 5b) across the 18 samples, which showed better sequencing converage and larger number of candidate lncRNAs than previous studies (Table 1). At the transcript level, we identified 4,589 lncRNA transcripts and 14,471 mRNA transcripts. Upon aligning these transcripts to the annotation database, we discovered 1,527 novel lncRNAs and 288 novel mRNAs (Fig. 6a). In terms of gene-level analysis, we identified 8,124 (SP vs CE), 80 (PP vs SP), 52 (AP vs PP), 49 (MP vs AP), and 137 (OP vs MP) differentially expressed lncRNAs between adjacent age periods. The numbers of differentially expressed lncRNAs between CE and each of the other groups were 7,470 (PP vs CE), 8,015 (AP vs CE), 5,226 (MP vs CE), and 8,352 (OP vs CE). Additionally, there were 2,152 (OP vs SP), 859 (OP vs PP), and 1,354 (OP vs AP) differentially expressed lncRNAs when comparing OP with the other groups (Fig. 6b). LncRNAs that showed differential expression profiles in one period compared to the other five periods were defined as period-specific lncRNAs. We identified 4,278 period-specific lncRNAs in CE, 8 in SP, 3 in PP, 1 in AP, 5 in MP, and 49 in OP, respectively. Collectively, there were more differentially expressed lncRNAs in the CE and OP groups compared to the other groups, suggesting a crucial role of lncRNAs in retinal development and aging.

**Table 1:** Comparison of lncRNA datasets of developing C57 mice retinas.

Article	Time points	Methods	Platform	No. of lncRNAs	Notes
Wan *et al*. BMC Genomics. 2019 Jul 8;20(1):559.	E12.5, E14.5, E16.5, E18.5, P0, P3, P5, P7, P14, P21 and P28	Full-length transcript sequencing	PacBio Sequel System	5404 lncRNAs (940 intergenic lncRNAs)	/
Chen *et al*. BMC Genomics. 2021 Oct 30;22(1):779.	E14.5, P1, P7, P12, P17, P56	Whole-transcriptome (circRNAs, lncRNAs, miRNAs and mRNAs) sequencing	Illumina HiSeq PE150	2600 lncRNAs, 13647 mRNAs, 27319 circRNAs, and 704 miRNAs	/
Yu *et al*. BMC Genomics. 2023 May 10;24(1):252.	E12.5, E13.5, E14.5, E15.5, E16.5, E17.5, E18.5, P0, P1, P3, P5, P7, P14, P21 and P28	Short-read sequencing	Illumina HiSeq PE150	4523 lncRNAs	Combined with secondary analysis of Wan *et al*. BMC Genomics. 2019 Jul 8;20(1):559.
Chen *et al*. Invest Ophthalmol Vis Sci. 2017 Dec 1;58(14):6308–6317.	P0, P56	LncRNA microarray	Affymetrix Mouse Transcriptome Array (MTA 1.0)	910 lncRNAs (P0) and 616 lncRNAs (P56)	Dataset encompassing six ocular tissues (cornea, lens, retina, RPE, choroid, and sclera)
This study	P5, P21, P42, P70, P180, P450	Integrated lncRNA and mRNA sequencing	Illumina HiSeq PE150	11222 lncRNAs and 18809 mRNAs	/

In gene-level analysis, we found 13,418 (SP vs CE), 129 (PP vs SP), 71 (AP vs PP), 67 (MP vs AP), and 235 (OP vs MP) differentially expressed mRNAs between adjacent age periods. The numbers of differentially expressed mRNAs between CE and each of the other groups were 12,245 (PP vs CE), 13,272 (AP vs CE), 8,525 (MP vs CE), and 13,988 (OP vs CE). Additionally, there were 3,603 (OP vs SP), 1,403 (OP vs PP), and 2,281 (OP vs AP) differentially expressed mRNAs when comparing OP with the other groups. We identified 6,935 period-specific mRNAs in CE, 10 in SP, 3 in PP, 3 in AP, 4 in MP, and 94 in OP, respectively.

### Functional annotation database enrichment analysis

**Fig. 7:**
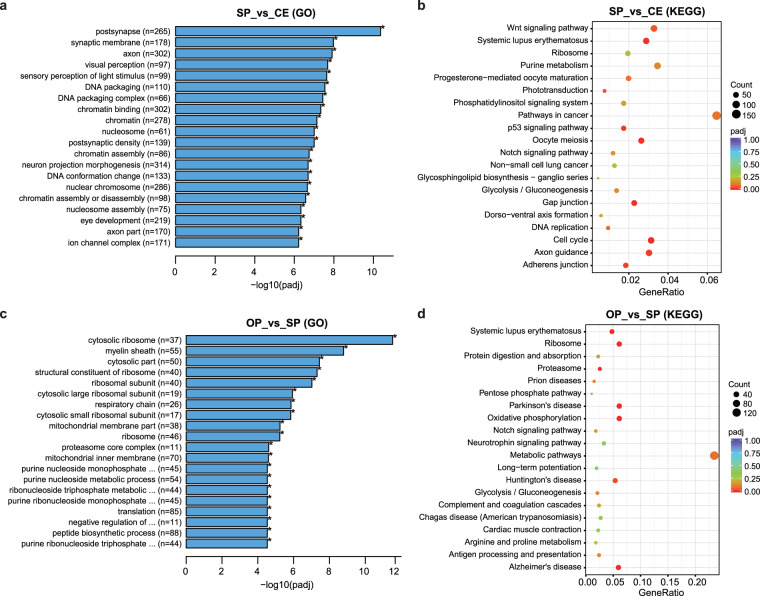
GO and KEGG analysis of differential expressed lncRNA/mRNA of SP vs CE and OP vs SP groups. (**a**) GO terms of SP vs CE might classify into light perception, retinal neuron structural development, DNA and chromatin events. **(b)** KEGG analysis of SP vs CE showed that phototransduction and axon guidance were enriched. **(c)** GO terms of OP vs SP mainly enriched in ribosome, mitochondrial activities, and purine metabolism. **(d)** KEGG analysis of OP vs SP showed that Parkinson disease, Alzheimer disease, metabolic pathways, oxidative phosphorylation were enriched. SP: suckling period; CE: close eye period; OP: old period.

GO analysis revealed that the top 20 enriched GO terms in the SP vs CE comparison could be classified into three main categories. The first category included terms related to light perception, such as visual perception, sensory perception of light stimulus, and eye development. The second category involved retinal neuron structural development, including terms like postsynapse, synaptic membrane, axon, postsynaptic density, neuron projection morphogenesis, and axon part. The third category encompassed DNA and chromatin events, including terms such as DNA packaging, DNA packaging complex, chromatin binding, chromatin, and nucleosome (Fig. 7a). KEGG pathway analysis demonstrated that the phototransduction pathway and axon guidance pathway were significantly enriched in the comparison between the SP and CE groups (Fig. 7b). These findings indicated that the epigenetic differences observed in lncRNAs and mRNAs between SP and CE were associated with phenotypic changes, including eye development, light perception, and retinal differentiation. The GO terms enriched in the comparison between the OP and SP groups were mainly related to ribosome, mitochondrial activities, and purine metabolism (Fig. 7c). KEGG pathway analysis revealed that the host genes in this comparison were primarily associated with Parkinson’s disease, Alzheimer’s disease, metabolic pathways, and oxidative phosphorylation (Fig. 7d). Additionally, KEGG analysis of OP vs PP, OP vs AP, and OP vs MP comparisons showed that the most significantly enriched pathways included oxidative phosphorylation as well as Parkinson’s and Alzheimer’s diseases. These results indicate that differentially expressed lncRNAs and mRNAs play a crucial role in the aging process of the retina and central nervous system. Furthermore, the CE and OP groups exhibited a more abundant transcriptomic information of lncRNAs and mRNAs compared to the other groups.

### Experimental validation of lncRNA-otx2os1

**Fig. 8:**
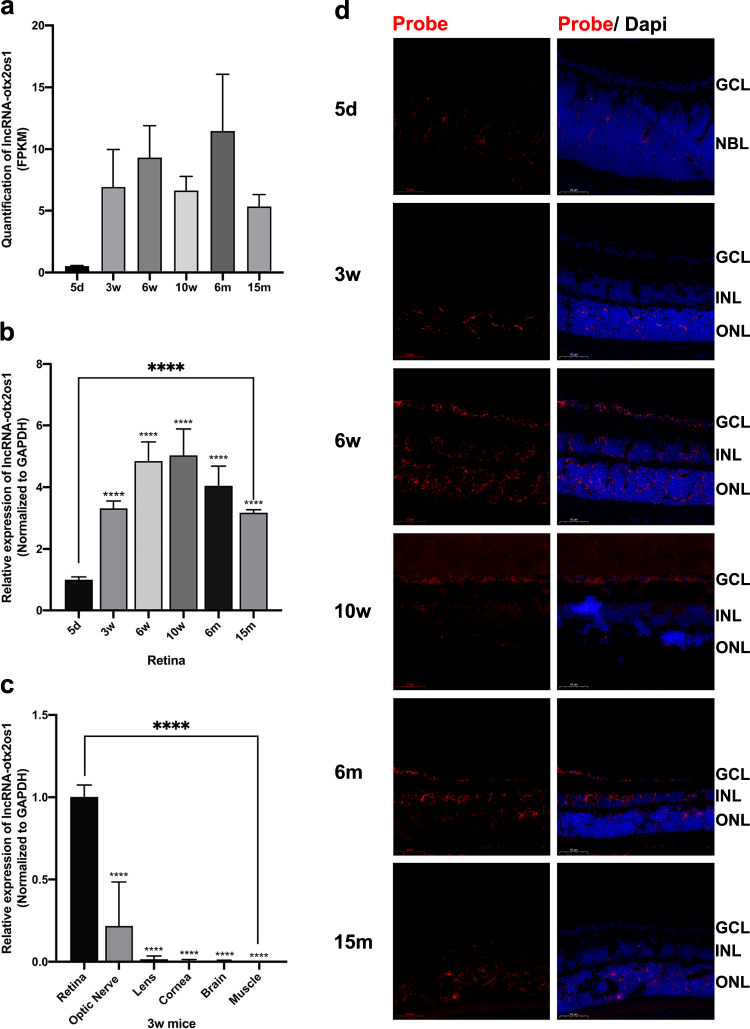
Laboratory validation of lncRNA-otx2os1 with potential of regulating retinal differentiation. **(a)** RNA sequencing and **(b)** qRT-PCR (ANOVA, n = 6, ****P < 0.001) showed similar expession patterns of lncRNA-otx2os1 in retina in six periods. **(c)** Results of qRT-PCR showed lncRNA-otx2os1 highly expressed in retina compared to other five tissues. **(d)** Fluorescence *in situ* hybridization showed that fluorescence intensity of lncRNA-otx2os1 corresponded with quantification in RNA sequencing. LncRNA-otx2os1 mainly located in neuroblast layer or outer nuclear layer, and increased in inner nuclear layer and retinal ganglion cell layer after differentiation and development. CE: close eye period; SP: suckling period; PP: puberty period; AP: adult period; MP: middle period; OP: old period; d: days; w- weeks; GCL: ganglion cell layer; NBL: neuroblast layer; INL: inner nuclear layer; ONL: outer nuclear layer.

After conducting a literature review and performing functional prediction, we selected lncRNA-otx2os1 (ENSMUST00000183522.8), one of the differentially expressed lncRNAs in the SP vs CE comparison, for experimental validation. Previous studies have shown that the host coding gene of lncRNA-otx2os1, Otx2, is involved in eye development and retinal differentiation^29–31^. The RNA sequencing results revealed that the expression of lncRNA-otx2os1 increased in the retina after eye opening, reached its peak during the adult/middle period, and decreased during the aging period (Fig. 8a). The qRT-PCR results further confirmed the developmental expression pattern of lncRNA-otx2os1 (Fig. 8b). Moreover, lncRNA-otx2os1 exhibited high tissue specificity in the retina compared to the optic nerve, lens, cornea, brain and muscle (Fig. 8c). The FISH results demonstrated that the fluorescence intensity of lncRNA-otx2os1 corresponded to its quantification in RNA sequencing. LncRNA-otx2os1 was predominantly located in the outer nuclear layer (ONL) and showed increased expression in the inner nuclear layer (INL) and RGC layer during differentiation and development (Fig. 8d). These experimental validation results of lncRNA-otx2os1 confirmed the reliability of the RNA sequencing data and served as an illustrative example of experimental validation of key lncRNAs.

## Usage Notes

This transcriptome dataset comprises comprehensive developmental and aging periods of mouse retinas, obtained from 18 samples with 3 biological replicates. The data generation and analysis processes were meticulously conducted, ensuring the high quality and reliability of the sequencing data. Notably, the CE and OP periods exhibited a greater abundance of lncRNA and mRNA transcriptomic information compared to other periods, reflecting retinal differentiation and aging, respectively. The utilization of this transcriptome dataset can unveil extensive gene and transcript expression information associated with retinal development, differentiation, homeostasis, and aging, thereby enhancing our understanding of physiological and pathological processes in the retina. Additionally, this dataset of normal mouse retinas can serve as a control group for mouse models of retinal diseases, presenting new targets for various retinal diseases, particularly age-related macular degeneration and retinitis pigmentosa. Furthermore, this bulk RNA sequencing dataset can serve as a reference for integrated analysis of transcriptional profiles in retinal single-cell sequencing, enabling the identification of cell-specific lncRNAs and mRNAs. Compared with previous studies of mouse retinal lncRNA sequencing with the oldest time point of 2 months, 15 months were the older time point which could capture aging efficiently (Table 1), but this dataset might be more valuable to add sequencing data at 21 months or older in future studies.

All raw RNA sequencing data are stored in FASTQ files, named as sample-1 or 2-fq.gz. The raw data for GO analysis, KEGG analysis, and differential expression analysis are stored in XLS files, which can be efficiently explored and analyzed using Microsoft Excel software.

### Supplementary information

Supplementary Figure 1

Supplementary Table1

Supplementary Table2

## Data Availability

The following open access software were used for quality control and data analysis as described in the main text: CASAVA, version 1.8.2, was used to convert raw image files into sequenced reads. HISAT2, version 2.0.5, was used to map reads to the reference genome. (http://daehwankimlab.github.io/hisat2/). StringTie, version 1.3.1, was used to assemble transcripts and calculate the numbers of transcripts mapped to the transcripts of database. (https://www.cnblogs.com/raisok/p/11046403.html). Cuffcompare, version 2.2.1, was used to align transcripts to annotated database. CNCI; CPC2, version 3.2.0; Pfam scan, version 1.3, were used to predict coding potential. EdgeR R package, version 3.12.1, was used to perform differential expression analysis of two conditions/groups. ClusterProfiler, version 4.6.2, was used to perform GO and KEGG pathway analysis. (http://www.bioconductor.org/packages/release/bioc/html/clusterProfiler.html).
