# Supplementary material for: Integrated Transcriptome Analysis of Long Noncoding RNA and mRNA in Developing and Aging Mouse Retina
Source: Sci Data. 2023 Sep 23;10:653. doi: 10.1038/s41597-023-02562-9 (PMC10518015; doi:10.1038/s41597-023-02562-9)
Supplement: Supplementary file 1 — Supplementary Figure 1 [file 41597_2023_2562_MOESM1_ESM.pdf]

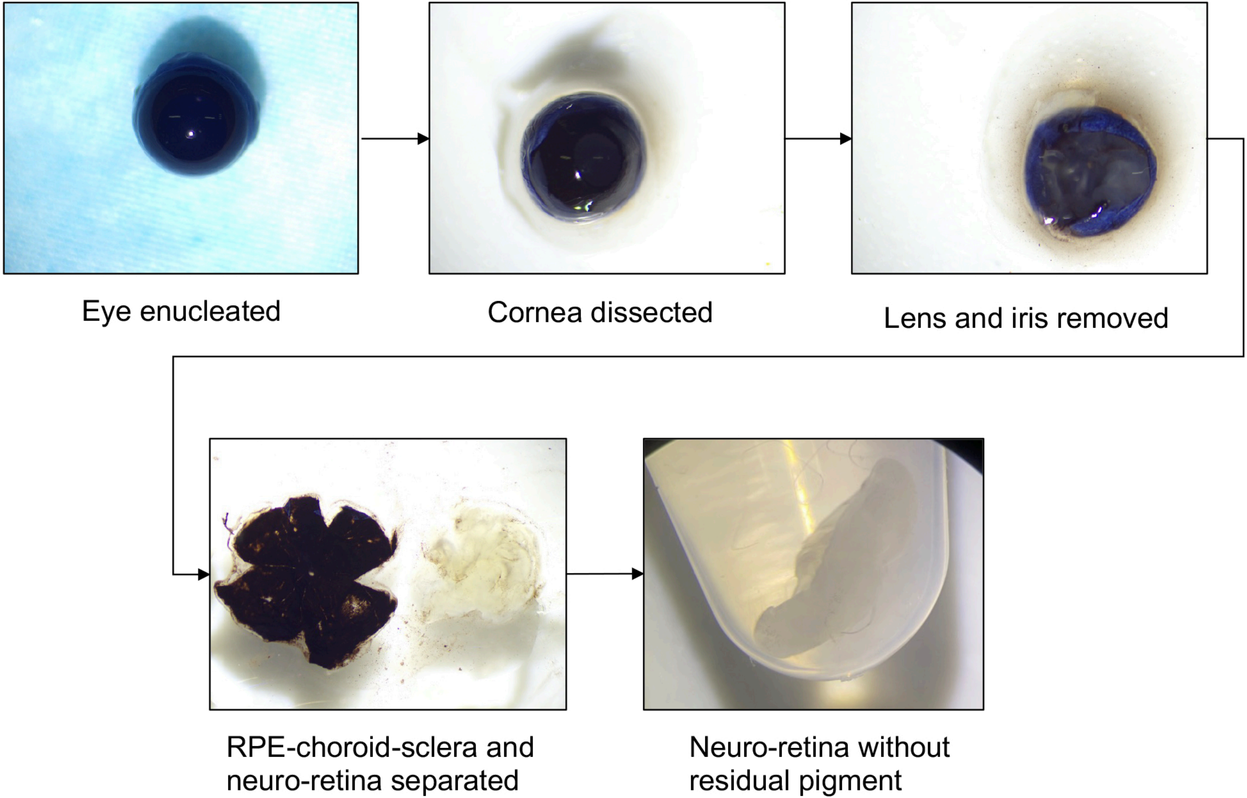
Eye enucleated
Cornea dissected
Lens and iris removed
RPE-choroid-sclera and neuro-retina separated
Neuro-retina without residual pigment

Supplementary Figure 1. Procedures of retina collection.
The eyes were enucleated and washed with PBS. Cornea was dissected along with limbus before removing lens and iris. Then neuro-retina was separated from RPE-choroid-sclera and washed with PBS to remove residual pigment.
